# Invertebrate diversity in groundwater‐filled lava caves is influenced by both neutral‐ and niche‐based processes

**DOI:** 10.1002/ece3.11560

**Published:** 2024-06-25

**Authors:** Bjarni K. Kristjánsson, Doriane Combot, Anett Reilent, Joseph S. Phillips, Camille A.‐L. Leblanc

**Affiliations:** ^1^ Department of Aquaculture and Fish Biology Hólar University Sauðárkrókur Iceland; ^2^ Department of Biology Creighton University Omaha Nebraska USA

**Keywords:** benthic, Cladocera, epibenthic, isolation, small populations

## Abstract

Understanding which factors shape and maintain biodiversity is essential to understand how ecosystems respond to crises. Biodiversity observed in ecological communities is a result of the interaction of various factors which can be classified as either neutral‐ or niche‐based. The importance of these processes has been debated, but many scientists believe that both processes are important. Here, we use unique ecosystems in groundwater‐filled lava caves near Lake Mývatn, to examine the importance of neutral‐ versus niche‐based factors for shaping invertebrate communities. We studied diversity in benthic and epibenthic invertebrate communities and related them to ecological variables. We hypothesized that if neutral processes are the main drivers of community structure we would not see any clear relationship between the structure of community within caves and ecological factors. If niche‐based processes are important we should see clear relationships between community structure and variation in ecological variables across caves. Both communities were species poor, with low densities of invertebrates, showing the resource limited and oligotrophic nature of these systems. Unusually for Icelandic freshwater ecosystems, the benthic communities were not dominated by Chironomidae (Diptera) larvae, but rather by crustaceans, mainly Cladocera. The epibenthic communities were not shaped by environmental variables, suggesting that they may have been structured primarily by neutral processes. The benthic communities were shaped by the availability of energy, and to some extent pH, suggesting that niche‐based processes were important drivers of community structure, although neutral processes may still be relevant. The results suggest that both processes are important for invertebrate communities in freshwater, and research should focus on understanding both of these processes. The ponds we studied are representative of a number of freshwater ecosystems that are extremely vulnerable for human disturbance, making it even more important to understand how their biodiversity is shaped and maintained.

## Graphical abstract

Here, we examine how ecological factors shape invertebrate diversity in ponds found in lava caves in Iceland. We specially focus on the importance of neutral and niche‐based processes.

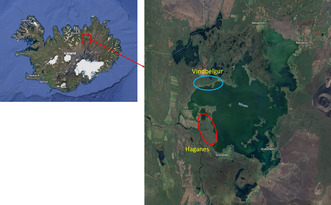

## 1 INTRODUCTION

The world's biodiversity is facing an accelerating, and unprecedented, decline (Secretariat of the Convention on Biological Diversity, 2020). This can be clearly seen in freshwater ecosystems which have been altered, fragmented, and simplified because of anthropogenic influences (Cairns & Lackey, 1992). Furthermore, changes in global climate have added to the biodiversity crisis (Pörtner et al., 2021). To be able to understand how ecosystems respond to crisis, it is essential that we understand which physical and ecological factors shape and maintain biodiversity, at all levels from genetics to communities and ecosystems.

The biodiversity observed in ecological communities is the result of the interaction of various factors, as has been discussed in relation to community assembly theory (Hutchinson, 1959; Vellend, 2010), where the observed community structure is the result of the interplay of four main processes; selection, ecological drift, speciation, and dispersal (Vellend, 2010). These processes can be classified as either neutral (stochastic) or niche‐based (deterministic) (Chase & Myers, 2011). The importance of neutral‐ versus niche‐based processes have been greatly debated (Chase & Myers, 2011; Clark, 2012; Gravel et al., 2011; Rosindell et al., 2012), but many scientists believe that both processes are important for community structure and dynamics (Gewin, 2006). Neutral processes are mainly related to dispersal ability of species, the order of colonizers into habitats and random factors related to species extinction (Hubbell, 2001; Vellend et al., 2014). Niche‐based processes relate to how the different species in the community use available resources and thus establish their niche (Van Valen, 1965; Vellend et al., 2014). Such a realized niche can be greatly influenced by various internal and external factors, for example, interactions among species within the community (e.g., competition and predation) and external environmental factors (e.g., temperature and food availability) (Ingram et al., 2018). Those external factors may subsequently influence species interactions. The importance of neutral‐ versus niche‐based factors can, for example, be studied by comparing communities in a group of similar, although distinct, habitats to variation in ecological factors across these habitats. Strong relationships between ecological factors and community structure are clearly indicative of the importance of niche‐based factors in shaping communities. The importance of neutral processes can be seen, for example, when community structure shows a clear gradient in relation to where the communities originate from (source habitat).

In all communities, energy demand and energy availability play a crucial role in shaping the community structure. The energy demand is strongly related to physiology, life‐history stage, and behavior of the species that make up the community (Brown et al., 2004). All these factors are further shaped through natural selection and phenotypic plasticity. Furthermore, in ectothermic species, environmental temperature is a key to the energy demand within the ecological community (Brown et al., 2004). Energy availability within communities is dependent on internal production, through photosynthesis and external input, both of which are commonly highly seasonal at higher latitudes (Dreyer et al., 2015).

**FIGURE 1:**
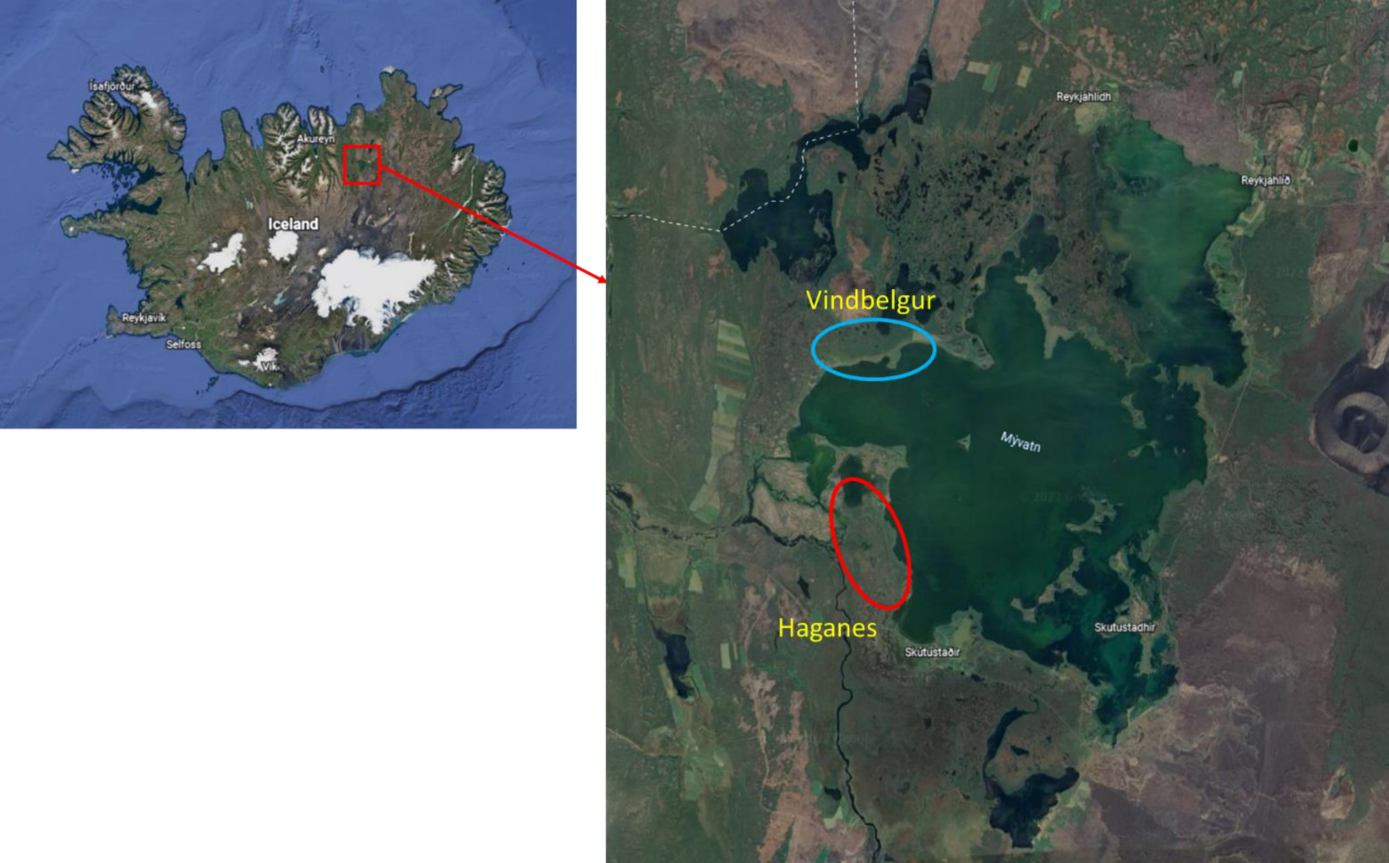
The location of the two areas, Vindbelgur and Haganes, with lava cave ponds studied for invertebrate communities around Lake Mývatn in Iceland.

Here, we study the invertebrate community structure in a number of small groundwater‐fed ponds, found in lava caves around Lake Mývatn, NE Iceland (Figure 1). The lava caves were created by a reduction of the volume of molten pahoehoe lava under a solidified crust originating in an eruption in the period 350–170 BCE (Hauptfleisch & Einarsson, 2012) which also created Lake Mývatn (Thorarinsson, 1951). The lava originated in an eruption. As these ponds are located in caves, photosynthesis is limited to various degrees and they are highly dependent on external inputs of organic material. Such inputs are temporally variable, both seasonally and across years, and consist mainly of adult chironomids (Leblanc & Kristjánsson, personal observation) that originate often in great numbers from the nearby Lake Mývatn, where populations may fluctuate over 3–4 orders of magnitude (Einarsson et al., 2004; Garðarsson et al., 1995; Ives et al., 2008). In addition, there is a clear gradient in the biomass of chironomid input to the ecosystem in relation to distance from Lake Mývatn (Dreyer et al., 2015). The ponds are found in two main areas, Vindbelgur and Haganes, which are fed by different groundwater flows. The ponds differ in size, physical factors of the pond water (e.g., temperature and oxygen availability), and distance from the lake, which is the most likely the source for invertebrates colonizing these habitats.

The cave ponds offer a good opportunity to examine the importance of neutral‐ versus niche‐based factors for shaping invertebrate communities. We thus hypothesize (1) that if neutral processes are the main drivers of community structure among ponds we cannot detect any clear relationship between community composition and ecological factors. However, (2) if niche‐based processes are important for the community structure we should see a clear relationship between community structure and variation in ecological variables across caves.

At the same time as we put forward these contrasting hypotheses, we are aware that it is likely a mixture of neutral‐ and niche‐based factors that may shape the communities.

## 2 METHODS

Sample collection for this project was done in collaboration with an ongoing monitoring project of fish, the small benthic Arctic charr (*Salvelinus alpinus*) inhabiting these caves (Figure 1, Leblanc et al., 2024), which to some extent dictated the methods used.

### 2.1 Environmental measurements

The following environmental variables in the caves were measured in June and August from 2013 to 2019: temperature (°C), oxygen saturation (%), pH, and specific conductivity (μS/cm), using a multiprobe sonde (Hydrolab DS5 (46711) Water Quality Probes). The invertebrate communities were sampled only in 2014 (see below), and in principle, we could have used environmental measurements from only 2014 in our analysis. However, niche‐based structuring of the communities likely unfolded over a longer time scale than a single year, and so a snapshot of the variables in 2014 may not have provided the best characterization of the typical conditions governing community structure in the caves. To investigate this further, we analyzed each environmental variable from 2013 to 2019 using linear mixed models to test for differences among caves, seasons, years, and associated interactions. In general, the environmental measurements varied among caves and through time. However, there were no significant cave × season or cave × year interactions, indicating that differences among the caves were consistent through time. Moreover, variation among years did not follow any obvious systematic trend. Therefore, we averaged the environmental variables across seasons and years from 2013 to 2019 for each cave to use for our subsequent analyses. We acknowledge that doing so entails comparisons of community structure to future environments. However, these averages provided the best characterization of typical conditions in the caves given the available data.

From a digital map, we estimated the minimal distance of each cave from the lake (in meters) and key physical characteristics of the caves, such as the combined vertical area of all openings and the size of the cave pond exposed to air (both in m^2^), in some caves, the roof was overhanging, so the pond exposed to air value was negative. Based on this, we estimated the area available for photosynthesis by adding half the vertical area of openings to the size of the cave pond exposed to air. This formula is an estimation and considers that high openings allow sunlight to reach further into the cave.

In each cave, we placed fall‐in traps within 20 cm of the shore to collect external inputs into the pond. The main objective of the traps was to estimate food availability for Arctic charr for another study. The traps were built of clear buckets (Unipak model 5141, 2300 mL, 196 cm^2^ surface area) placed in a bracket and tied fast with a zip‐tie about 10 cm above the water surface. In each bucket, we mixed propylene glycol (30%) with water and a few drops of aroma‐free soap to reduce water tension. The number of traps differed among the caves, depending on the size of the cave and the number of cave openings, but the minimum number of fall‐in traps per cave was two. However, not all traps survived the conditions during the winter, so on some occasions no trap data exist for a given sampling event. The traps were collected in June and August each year, by removing the old trap and replacing it with a new one. The content of the fall‐in trap was sieved through a 125 μm sieve and preserved in 70% ethanol for at least 6 months. The fixed organic material of animal origin was then separated from obvious plant material, placed in a measuring cup, and allowed to sink for 15–30 min, and measured to the nearest mL. We thus obtained an estimation of gross volume of external organic animal material per cave.

For each cave in each month (June or August), we averaged the volume of organic material from 2013 to 2016, with a similar rationale to above for characterizing typical conditions in the caves. This was done to make sure that in all caves, we had at least one sampling during the summer months and once during the winter months (see also discussion above). We then calculated the amount of organic material falling in the traps for each month (2 months between June and August, and 10 months between August and June). Lastly, we estimated the average amount of organic material (in mL per m^2^) falling each month on the air‐exposed surface in each pond. Caves with negative values for cave ponds exposed to the air, were set to as having exposed area of 0.02 m^2^ (the size of the bucket) surface area exposed to sky to prevent having 0 as a multiplier in the calculations.

### 2.2 Benthic and epibenthic invertebrates sampling

All invertebrate samples were collected in June 2014. Benthic invertebrates were collected from two to three stones along the shore of 16 caves, at a depth of 10–30 cm. Stones were carefully removed from the bottom and placed immediately in a 10 L bucket containing water from the cave. They were then brushed, using a soft dishwasher brush. The stone was rinsed with clean filtered (125 μm sieve) water, placed as they were lying in the cave and photographed with a mm scale for size reference. This allowed us to estimate standardized densities (#/100 cm^2^) of invertebrates across stones and caves. The water and invertebrates retained in the bucket were sieved through a 125 μm sieve and stored in 70% ethanol until further analysis.

Epibenthic invertebrates were collected from 18 caves using crustacean traps modified from Örnólfsdóttir and Einarsson (2004). The traps were haphazardly laid within 2 m from the shore at the opening of each cave. The number of traps per cave varied from two to six depending on the size of the cave and the size and number of openings. The traps were composed of a Plexiglas plate (140 mm × 145 mm × 4 mm), four carriage bolts (100 mm long) as legs, maintained by 10 mm nuts, 15 mm flat washers, and four container lids (95 mm) as feet to prevent the legs sinking into the sediment. A hole of 80 mm was cut out in the center of the plate to let a funnel through. The sampling containers were transparent plastic buckets (UniPak model 5110, 365 mL and 95 mm) affixed upside down on the Plexiglas frame and held together with a 5‐mm‐thick rubber band. Each container had a lid (UniPak ref. 281.0) with a hole drilled in the center to allow a funnel to slide in and be fixed with a 5 mm rubber band. The funnels (8 mm at the narrow opening and 75 mm at the wide opening) were shortened from 130 mm to 95 mm to allow a space between the bottom of the container and the end of the funnel. Once deployed, the wide opening of the funnel sits about 30 mm above the sediment surface. Prior to laying the traps, the containers were filled up with filtered water (63 μm mesh) collected from the cave. This was done to prevent any non‐filtered water from seeping in when deployed. The traps were collected after 12 h, and their content filtered through a 63 μm sieve and stored in 70% ethanol until further analysis.

In the laboratory, the samples, both benthic and epibenthic, were sorted and all invertebrates picked out. Individuals of terrestrial origin (mostly adult blackfly *Simulium vittatum*) were removed from the samples. Individuals were then identified to the finest practical level of taxonomic resolution (see Section 3).

### 2.3 Statistical analysis

All statistical analyses were conducted in R (version 4.2.2., R Core Team, 2022) using the packages specified below. Variation in environmental variables across areas (H and V) and caves was examined using Welch's *t*‐tests for between area comparisons and Pearson correlations were used for between ecological variables. The amount of organic material was highly correlated with the area available for photosynthesis. Because of this, we included only the amount of organic material as a proxy of production in the caves as factor in the subsequent analyses.

The same statistical analysis was conducted for both stone samples and epibenthic traps. We reported the number of taxa within each cave in addition to the Shannon diversity, using the *diversity* function in the *vegan* package (Oksanen et al., 2022). These indexes were then compared to environmental variables using multiple regression performed with the *lm* function.

We transformed the data for further analysis. We first log_10_ transformed density/catch data for each taxon, which was followed by a *Z* transformation of both invertebrate and environmental variables, where each measurement was standardized by subtracting the mean from the variable and divide it by the standard deviation. This was done to normalize and standardize the data.

We summarized the community diversity across caves using non‐metric multidimensional scaling (NMDS) based on Bray–Curtis distances on mean transformed values of all stones within each cave, using the function *metaMDS* in the package *vegan*. We visualized how ecological factors (which had significant relationships) related to variation in invertebrate communities by visualizing results using the function *envfit* in the package *vegan*. To complement this ordination analysis, we used two statistical methods for inferring variation in community composition in association with the environmental variables. First, we used a PERMANOVA fit using the *adonis2* from the *vegan* package, with a Bray–Curtis dissimilarity matrix and 20,000 permutations. This method provides a synoptic assessment of how community composition as a whole varied due to the environmental variables.

Second, we used linear mixed models (LMMs) to quantify variation in the taxon‐specific responses to the environmental variables (Jackson et al., 2012). We fitted the models using the function *lmer* from the package lme4 (Bates et al., 2015), with the stones/traps nested within caves as a random factor. For this analysis, the data were converted to a “long” format, with a single variable representing the density of each invertebrate group on each stone/trap in each cave (“Invertebrate density”) and a corresponding variable indicating the taxon to which a given observation belongs (“Invertebrate group”). This resulted in 1248 observations for the stones and 912 for the traps. The full model was then fit with the following structure: Invertebrate density ~ Temp + Area + pH + Oxygen + Cond + Distance + Organic matter + (Temp + Area + pH + Oxygen + Cond + Distance + Organic matter | Invertebrate group) + (1 | Cave/stone (or trap)). The fixed effects corresponded to overall responses across all taxa, while the random effects term grouped by “Invertebrate group” captured the variation among taxa in their responses to the environmental variables. When taxa differ in their abundance responses to environmental variation, this in turn implies that the composition of the community also changes. Therefore, the LMM provided a similar inference to the PERMANOVA regarding variation in community composition but went further by providing information on how each taxon responded to the environmental variables (Jackson et al., 2012).

When using LMMs, we determined significance of the fixed effects (i.e., the overall response across all taxa) of the ecological variables with the *Anova* function from the package Car (Fox & Weisberg, 2019). We then determined whether the ecological variables corresponded with changes in community composition by examining random effects that quantified variation in responses among different taxa. Specifically, we compared the fit of the full model to a reduced model removing the random effect associated with a given environmental variable grouped by taxon using likelihood‐ratio tests implemented with the *anova* function. Finally, we examined the response of each taxon to the ecological predictors using the individual random effects levels estimated when fitting the LMMs. We quantified standard errors for these random effects levels through parametric bootstrapping using the function *bootMer* in the package lme4.

## 3 RESULTS

### 3.1 Environmental samples

Overall, the averaged environmental variables were similar across caves and temperature ranged by 1.9°C, pH was alkaline and ranged by 2.1, and conductivity was relatively low ranging from 97 in Cave 19 to 142 in Cave 12 (Table 1). Caves ranged from 29.2% in Cave 6 to 70.7% in Cave 17 in oxygen saturation. The caves differed considerably in the area available for photosynthesis and the amount of terrestrial organic matter. The distance between the caves and the lake ranged from 57 to 406 m (Table 1).

**TABLE 1:** Environmental variables measured in Lava caves around Lake Mývatn, Iceland.

Cave	Area	Temp	pH	Cond	Oxygen	Photosynthesis	Distance	Organic matter
1	H	7.6	8.8	141	52.3	4.6	170	14.4
2	H	5.3	8.9	136	42.9	14.7	174	5.9
5	H	7	8.1	128	46.9	7.7	110	6.9
6	H	6.7	8.3	133	29.2	1.9	102	1.9
7	H	6.4	8.9	141	33	21.2	117	68.9
10	H	7	8.4	134	51.2	12.9	166	8.4
11	H	6.7	8.3	127	44.3	7.1	139	5.3
12	H	7.8	7.1	142	31.4	10.7	90	9.3
17	V	6	9.2	99	70.7	2.5	378	3.8
18	V	6.3	8.8	101	68.5	19.6	406	57.0
19	V	6.1	8.8	97	61.4	6.8	235.5	31.6
20	V	6.2	8.8	98	62.3	9.2	172	12.8
21	V	7.2	8	104	44.9	16.6	61	276.6
22	V	6.7	8.5	103	45.5	10.3	110	87.5
23	V	6.8	7.8	111	41.7	5.2	174	25.9
25	H	6.7	8.9	140	36.3	59.2	139	354.0
27	H	6.5	7.8	111	46.8	15.5	57	25.2
17B	V	6	8.9	98	59.1	16.5	370	73.8

*Note*: The table shows, the area the cave is found in, either Vindbelgur (V) or Haganes (H), average temperature (°C), pH, conductivity (μS/cm), oxygen saturation (%), area for photosynthesis (m^2^), minimal distance to Lake Mývatn (m) in a straight line, and the average mL of organic matter of terrestrial origin per month per m^2^ of surface area of the cave pond exposed to air.

**FIGURE 2:**
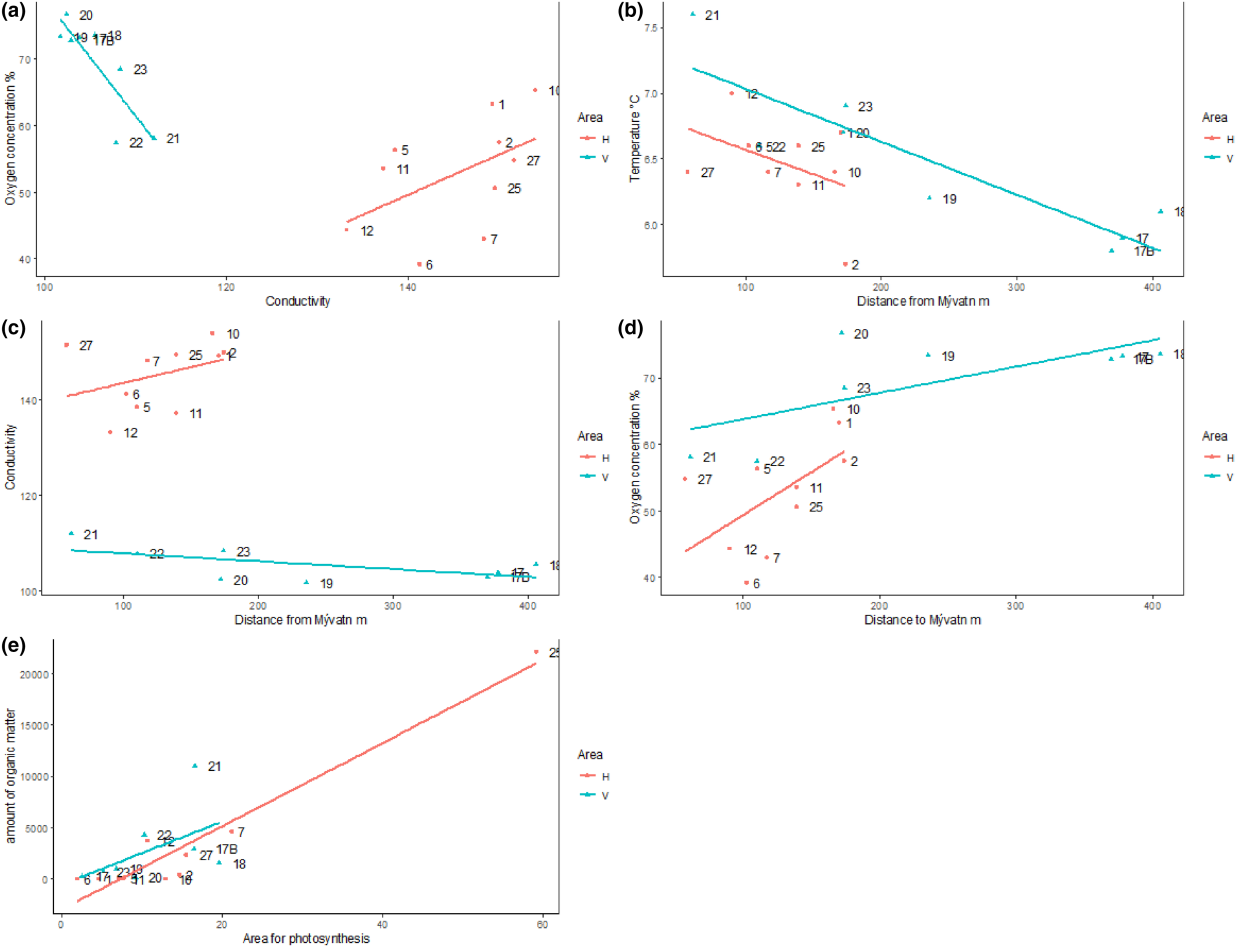
Relationships between environmental factors of lava caves from two areas, Haganes (H) and Vindbelgur (V) around Lake Mývatn. (a) Relationship between conductivity and oxygen concentration. (b) Relationship between distance from Lake Mývatn and temperature. (c) Relationship between distance from Lake Mývatn and conductivity. (d) Relationship between distance from Lake Mývatn and oxygen concentration. (e) Relationship between the average amount of organic matter (in mL) falling on the opening of each cave monthly and the area of cave surface available for photosynthesis.

Conductivity (*t*_(13.6)_ = 9.33, *p* < .001) and oxygen saturation (*t*_(12.7)_ = −3.2, *p* = .007) differed between the two areas, and these two variables were negatively correlated with each other (*r* = −.72, *t*_(16)_ = −4.18, *p* = .001; Figure 2a). Furthermore, the minimum distance to the lake had a negative correlation with temperature (*r* = −.50, *t*_(16)_ = −2.30, *p* = .035; Figure 2b) and conductivity (*r* = −.50, *t*_(16)_ = −2.28, *p* = .036; Figure 2c), but a positive correlation with concentration of oxygen (*r* = .78, *t*_(16)_ = 4.96, *p* < .001; Figure 2d). The strongest observed correlation was between the area exposed for photosynthesis and the amount of organic matter (*r* = .81, *t*_(16)_ = 5.53, *p* < .001; Figure 2e). Other pairwise comparisons among environmental variables did not reveal statistically meaningful patterns.

### 3.2 Benthic invertebrates

**FIGURE 3:**
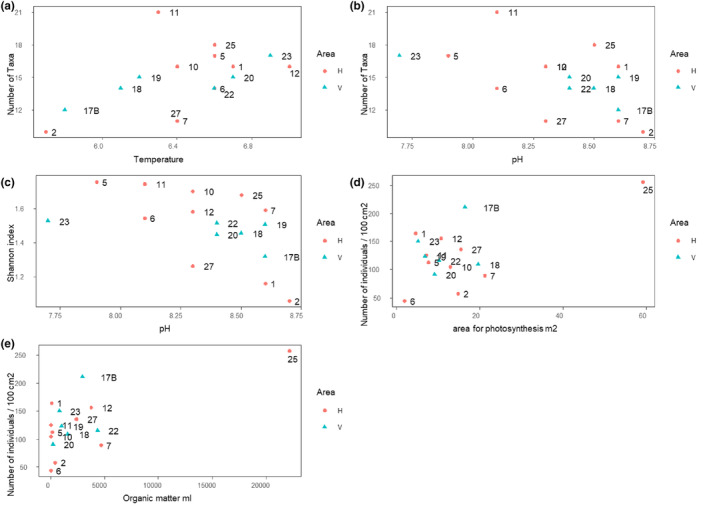
Relationships between environmental factors and alpha diversity of invertebrate communities on hard bottom in lava cave ponds. Caves from the two areas are shown, Haganes (H) in red and Vindbelgur (V) in blue. (a) Temperature and taxa richness. (b) pH and taxa richness. (c) pH and Shannon diversity. (d) Area available for photosynthesis and density of invertebrates. (e) Average amount of organic matter (in mL) falling on the opening of each cave monthly and density of invertebrates.

A total of 26 taxa were found on the stones in the caves (Table 2). Copepoda, Ostracoda, *Chyodorus sphaericus* (Cladocera), and the midge subfamily Orthocladiinae (Chironomidae) were found in all the caves, and freshwater mites (Acariformes) in all but one (cave 7). Other groups were found in fewer caves, with the *Sida crystallina* (Cladocera) and the *Chaetogaster* sp. (Clitellata) only found in cave 11. The average number of taxa found in the caves was 15, with the fewest (10) found in cave 2 and the highest (21) in cave 11. The average density of invertebrates per 100 cm^2^ was 127.97 (±53.3 SD calculated among caves after averaging values for stones). The highest density was seen in cave 25 (257.3 ± 67.80) and the lowest in cave 6 (44.1 ± 26.63), which happen to be the most and least exposed areas for photosynthesis, respectively. On average, Copepoda had the highest densities (42.2 ± 25.7) followed by Ostracoda (34.5 ± 20.0) and Nematoda (14.5 ± 14.2). In all cases, there was considerable variation among stones within each cave as seen in high standard deviations. Shannon diversity was quite similar across the caves, with the lowest value being 1.32 in cave 17B and the highest value being 1.76 in cave 5. In general, the number of taxa, invertebrate density, and Shannon diversity did not differ between areas or vary across environmental predictors. The one exception is invertebrate density, which increased with organic matter (*t*_(8)_ = 3.75, *p* = .006, Figure 3).

**TABLE 2:** Average densities (#/100 cm^2^) of benthic invertebrates with standard deviation, Shannon index, and number of taxa in 16 Lava Caves near Lake Mývatn in Iceland.

Cave	Acarina	Aranea	Chaet	Chiro	Ortho	Tanyp	Tanyt	Clado	Cspha	Alona	Anana	Aharp
1	0.6 ± 1.06	0.3 ± 0.53	0.0	0.0	12.8 ± 6.87	0.0	0.0	0.2 ± 0.38	9.1 ± 7.84	0.0	0.0	0.0
2	0.8 ± 0.35	0.0	0.0	0.0	3.9 ± 6.13	0.0	0.0	0.0	11.6 ± 17.79	0.0	0.0	0.1 ± 0.14
5	0.7 ± 0.65	0.0	0.0	0.2 ± 0.37	1.2 ± 0.53	0.0	1.4 ± 1.29	4.0 ± 3.03	2.0 ± 1.22	6.0 ± 6.6	5.6 ± 9.77	1.4 ± 2.44
6	0.4 ± 0.71	0.4 ± 0.62	0.0	0.0	1.8 ± 2.10	0.0	0.2 ± 0.31	2.0 ± 1.38	1.6 ± 0.74	3.3 ± 3.78	0.0	2.4 ± 1.54
7	0.0	0.2 ± 0.32	0.0	0.0	8.9 ± 8.37	0.0	0.0	4.1 ± 7.14	1.0 ± 0.93	0.0	0.0	8.3 ± 4.02
10	1.3 ± 1.01	1.0 ± 1.69	0.0	1.5 ± 1.47	6.2 ± 6.62	0.7 ± 0.62	1.0 ± 1.69	3.2 ± 4.28	16.1 ± 18.34	20.7 ± 9.43	1.0 ± 1.11	5.8 ± 5.61
11	1.8 ± 1.07	0.2 ± 0.39	0.2 ± 0.39	0.2 ± 0.39	2.2 ± 1.20	0.0	3.0 ± 0.88	1.9 ± 1.07	1.2 ± 1.17	2.8 ± 2.02	0.0	4.4 ± 3.81
12	1.2 ± 0.25	0.0	0.0	0.0	3.5 ± 1.41	0.0	0.4 ± 0.36	3.5 ± 0.89	17.1 ± 12.43	11.4 ± 3.39	0.2 ± 0.41	0.5 ± 0.83
18	1.0 ± 1.14	0.0	0.0	0.0	6.5 ± 4.11	0.0	0.0	1.3 ± 1.14	1.3 ± 1.14	0.0	0.0	0.0
19	0.9 ± 0.78	0.0	0.0	0.0	13.0 ± 19.41	0.5 ± 0.46	0.6 ± 1.02	1.0 ± 1.78	3.3 ± 3.47	0.0	0.1 ± 0.25	2.5 ± 2.16
20	1.4 ± 1.61	0.0	0.0	0.0	1.3 ± 1.16	1.3 ± 1.66	0.6 ± 1.09	11.2 ± 16.78	1.2 ± 0.61	0.0	0.0	11.0 ± 10.98
22	1.7 ± 2.12	0.0	0.0	0.3 ± 0.55	8.4 ± 6.76	0.3 ± 0.47	0.0	9.2 ± 4.25	2.6 ± 2.75	3.4 ± 1.74	0.5 ± 0.94	0.8 ± 0.74
23	1.0 ± 1.45	0.0	0.0	0.3 ± 0.52	2.8 ± 1.87	0.0	0.0	1.2 ± 0.61	5.3 ± 3.75	0.8 ± 0.90	4.0 ± 3.41	6.6 ± 4.99
25	2.3 ± 2.01	0.0	0.0	0.2 ± 0.39	1.1 ± 1.92	0.9 ± 1.05	1.2 ± 1.1	14.1 ± 12.28	40.8 ± 50.51	11.3 ± 9.09	0.0	2.1 ± 1.06
27	3.2 ± 2.01	0.3 ± 0.53	0.0	0.0	6.9 ± 10.44	0.0	0.0	22.3 ± 38.57	53.3 ± 47.31	0.0	0.8 ± 1.43	3.8 ± 1.74
17B	0.6 ± 1.03	0.0	0.0	0.0	6.0 ± 2.90	0.2 ± 0.39	0.6 ± 0.63	0.2 ± 0.39	1.6 ± 1.82	0.0	0.9 ± 1.58	0.0

Cave	Mhirs	Isord	Scryst	Cole	Colle	Cope	Hydra	Nema	Ostra	Oligo	Pleco
1	0.2 ± 0.38	0.2 ± 0.38	0.0	0.0	0.9 ± 1.58	99.1 ± 51.87	0.0	14.0 ± 24.23	19.0 ± 27.43	5.1 ± 5.44	0.0
2	0.0	0.0	0.0	0.0	0.0	31.2 ± 21.16	0.0	0.8 ± 1.37	5.2 ± 5.28	4.2 ± 2.75	0.0
5	0.3 ± 0.49	0.0	0.0	0.0	0.0	37.9 ± 47.01	0.0	2.8 ± 2.59	43.6 ± 63.37	2.0 ± 3.42	0.3 ± 0.49
6	0.4 ± 0.71	0.0	0.0	0.0	0.8 ± 0.67	21.7 ± 15.30	0.0	0.4 ± 0.71	9.5 ± 6.97	0.0	0.0
7	0.0	0.0	0.0	0.0	0.5 ± 0.46	23.1 ± 12.61	0.0	0.0	33.7 ± 10.10	5.6 ± 4.47	0.0
10	0.5 ± 0.45	0.0	0.0	0.2 ± 0.36	0.0	36.0 ± 20.83	0.0	1.8 ± 1.70	11.2 ± 11.92	0.0	0.0
11	0.3 ± 0.34	0.2 ± 0.39	0.2 ± 0.39	0.0	0.1 ± 0.20	44.0 ± 14.00	0.0	30.8 ± 37.10	19.6 ± 8.38	11.2 ± 10.13	0.0
12	0.2 ± 0.41	0.0	0.0	0.0	0.4 ± 0.33	45.8 ± 6.26	0.0	5.5 ± 1.67	63.7 ± 5.39	1.4 ± 2.49	0.0
18	2.0 ± 0.54	0.0	0.0	0.0	0.5 ± 0.90	32.4 ± 29.58	0.0	7.1 ± 9.28	49.9 ± 59.12	5.4 ± 8.76	0.7 ± 1.28
19	2.1 ± 2.81	0.0	0.0	0.0	0.0	43.1 ± 31.87	0.0	9.5 ± 10.10	38.9 ± 18.51	6.1 ± 5.50	0.0
20	0.4 ± 0.39	0.0	0.0	0.0	1.9 ± 1.67	35.2 ± 22.59	0.2 ± 0.36	15.1 ± 13.62	9.2 ± 8.40	0.2 ± 0.36	0.0
22	0.3 ± 0.47	0.0	0.0	0.0	0.0	16.8 ± 19.13	0.0	14.3 ± 16.97	56.2 ± 31.74	0.0	0.0
23	0.0	0.0	0.0	0.0	0.3 ± 0.52	15.0 ± 7.00	0.3 ± 0.46	82.1 ± 67.31	20.1 ± 12.05	9.5 ± 6.52	0.0
25	0.0	0.2 ± 0.32	0.0	0.6 ± 0.96	0.2 ± 0.39	68.2 ± 33.94	0.0	30.7 ± 26.06	73.8 ± 7.39	6.6 ± 10.05	0.0
27	0.0	0.0	0.0	0.0	0.0	53.9 ± 50.63	0.0	0.0	11.2 ± 7.88	2.6 ± 1.00	0.0
17B	1.4 ± 2.37	0.0	0.0	0.0	0.0	71.3 ± 27.95	0.0	17.2 ± 14.89	87.6 ± 35.38	23.4 ± 20.22	0.0

Cave	Pupa	Tardi	Tricho	Density	Shannon	Taxa #
1	0.5 ± 0.47	1.9 ± 2.81	0.2 ± 0.38	164.2 ± 72.26	1.16 ± 0.182	16
2	0.0	0.0	0.0	57.7 ± 31.25	1.06 ± 0.164	10
5	2.2 ± 1.41	1.1 ± 1.95	0.0	112.7 ± 136.74	1.76 ± 0.194	17
6	0.0	0.3 ± 0.48	0.0	44.1 ± 26.63	1.54 ± 0.189	14
7	0.0	0.0	7.9 ± 5.42	89.1 ± 35.11	1.59 ± 0.034	11
10	0.0	0.0	0.0	104.9 ± 20.75	1.70 ± 0.164	16
11	0.5 ± 0.71	0.3 ± 0.51	0.0	125.4 ± 68.35	1.74 ± 0.266	21
12	0.2 ± 0.27	0.0	1.4 ± 1.25	156.4 ± 11.00	1.58 ± 0.126	16
18	0.2 ± 0.39	0.0	0.7 ± 1.28	109.0 ± 102.46	1.45 ± 0.111	14
19	0.2 ± 0.40	0.0	1.5 ± 1.40	123.4 ± 55.55	1.51 ± 0.137	15
20	0.4 ± 0.73	0.0	0.0	90.8 ± 42.36	1.45 ± 0.370	15
22	0.2 ± 0.28	0.0	0.0	115.0 ± 46.98	1.52 ± 0.185	14
23	0.3 ± 0.44	0.3 ± 0.44	0.3 ± 0.44	150.0 ± 41.73	1.53 ± 0.585	17
25	1.2 ± 1.11	0.0	1.7 ± 1.43	257.3 ± 67.80	1.68 ± 0.230	18
27	0.0	0.3 ± 0.50	0.0	136.28 ± 88.72	1.26 ± 0.175	11
17B	0.0	0.0	0.0	211.1 ± 94.37	1.32 ± 0.169	12

*Note*: Some taxa names have been shortened: Chaet (Chaetogaster), Chiro (Choronomidae sp.), Ortho (Orthocladiinae), Tanyp (Tanypodinae), Tanyt (Tanytarsini), Clado (Cladocera sp), C.spha (*Chyodorus sphaericus*), Anana (*Alonella nana*), Aharp (*Acroperus harpae*), Mhirs (*Macrothrix hirsuticornis*), Isord (*Ilyocryptus sordidus*), Scryst (*Sida crystallina*), Cole (Coleoptera), Colle (Collembola), Cope (Copepoda), Nema (Nematoda), Ostra (Ostracoda), Oligo (Oligochaeta), Pleco (Plecoptera), Tardi (Tardigrada), Tricho (Trichoptera).

**FIGURE 4:**
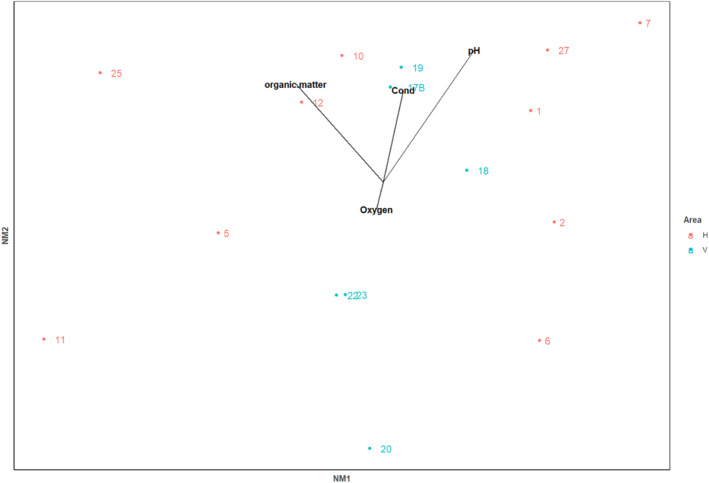
NMDS ordination showing the relationship among invertebrate communities on hard bottom in lava cave ponds. Caves from the two areas are shown, Haganes (H) in red and Vindbelgur (V) in blue. Environmental variables found to significantly relate to the invertebrate communities are overlaid.

**FIGURE 5:**
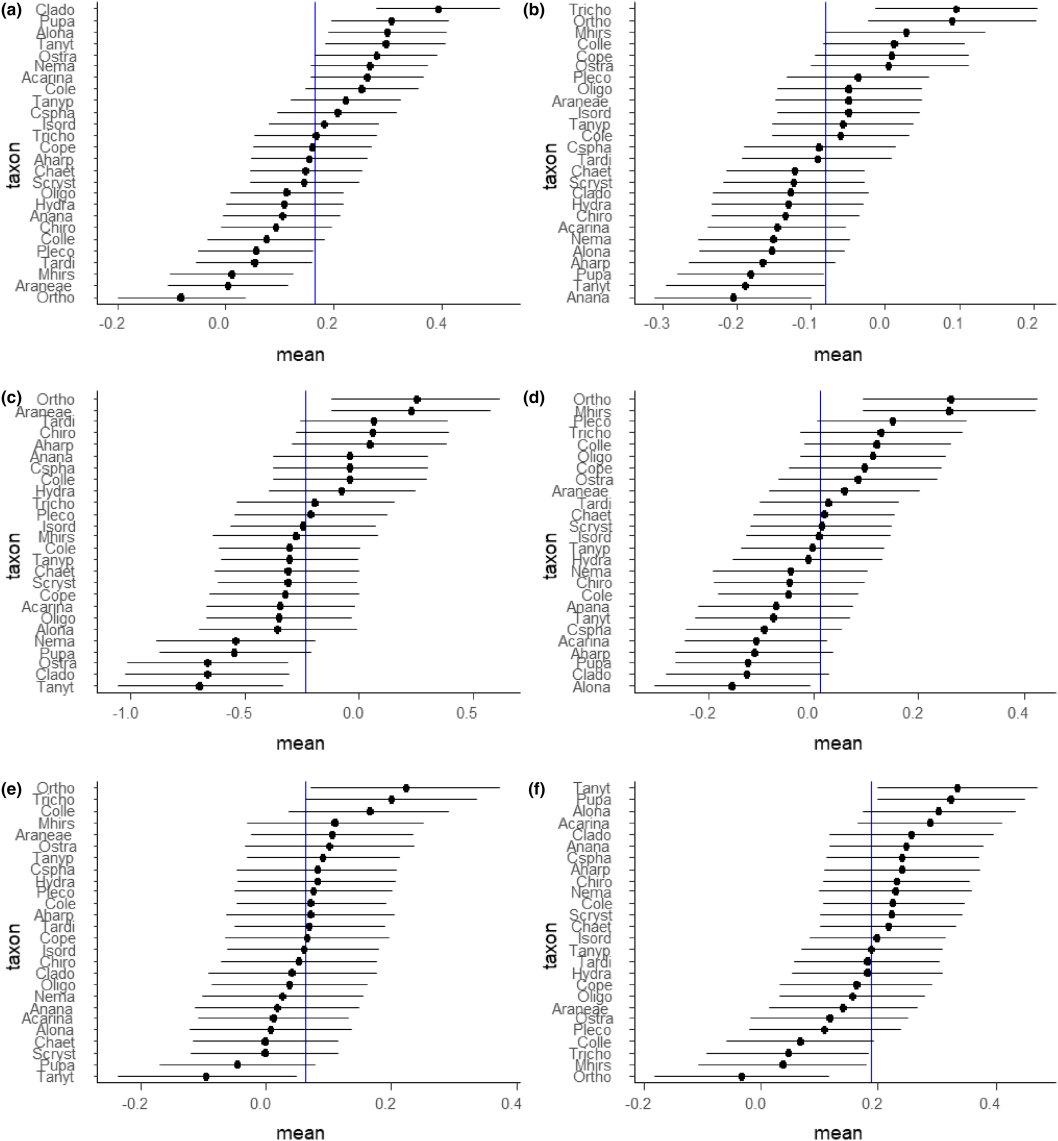
Mean responses, with standard error, of different taxa from invertebrate communities on hard bottom in lava cave ponds in relation to the average fixed effect estimate from a linear mixed model studying the effect of environmental variables on invertebrate communities with stones nested within caves as a random factor. The responses were obtained by parametric bootstrapping. The environmental variables are as follows: Organic matter (a), pH (b), conductivity (c), distance to Lake Mývatn (d), temperature (e), and oxygen concentration (f). Abbreviations for species are explained in Table 2.

The simplest solution for NMDS analysis was five dimensional, which gave a stress of 0.049. Plotting the first two axes (two‐dimensional stress—0.18) (Figure 4) showed considerable overlap of invertebrate communities of the caves from the two areas. However, there was much less variation in the caves in V on NMDS axis 1. According to the PERMANOVA, community composition varied the most strongly with the amount of organic matter (*F*_(1.40)_ = 2.3, *p* = .02, *R* ^2^ = .05), followed by pH (*F*_(1.40)_ = 1.7, *p* = .03, *R* ^2^ = .03), and conductivity (*F*_(1.40)_ = 1.6, *p* = .03, *R* ^2^ = .03). The importance of organic matter was reinforced by the LMM, which showed both consistently positive effects across taxa (*F*_(1,13.6)_ = 3.8, *p* = .07) and variation in taxon‐specific responses (*p* = .03) to organic matter. The abundances of unidentified Cladocera, *Alona* spp. (Cladocera), *Chydorus sphaericus* (Cladocera), Coleoptera, and freshwater mites were all higher in the presence of elevated organic matter (Figure 5a). In contrast, *Macrothrix hirsuticornis* (Cladocera) and Orthocladiinae (Chironomidae) had negative responses to organic matter. Unlike for the PERMANOVA, evidence of community composition changes in response to pH and conductivity was more equivocal from the LMM, both being far from being significant. It is worth noting that the PERMANOVA did not account for the grouping of observations within caves and therefore was likely to be less conservative than the LMM. However, it does seem that there was meaningful variation in response to pH, with *M. hirsuticornis* having a positive response and unidentified cladocera having a negative response to elevated pH (Figure 5b). The responses of groups to the other environmental variables, conductivity, distance from Lake Mývatn, temperature, and oxygen can be seen in Figure 5c–f.

### 3.3 Epibenthic invertebrates

A total of 12 taxa were found in the epibenthic traps, most of those being Cladocera (Table 3). Furthermore, 15 species of Ostracoda have been identified from these samples (Alalaj et al., 2019), although here we group them as Ostracoda. In Alalaj et al. (2019) detailed identification was not done in all caves of the present study. *Chydorus sphaericus* (Cladocera) was found in all the caves along with Copepoda and Rotifera. Those groups had by far the highest number of individuals caught (7.0, 29.2, and 45.6 on average across all caves, respectively). All other groups had on average less than one individual per sample. Within caves, the number of taxa ranged between 4 and 7 (Table 3). The highest average number of individuals in traps was found in cave 18 (323), but the lowest in cave 2 (26.7). There was, however, considerable variation among the caves, seen in high standard deviations. Average Shannon diversity was low but similar across the caves, with the lowest being 0.50 in cave 10 and the highest one being 1.1 in cave 21 (Table 3). In general, the number of taxa, number of individuals, and Shannon diversity did not differ between the two areas, nor did they vary across most of the environmental variables. However, Shannon diversity had a marginally positive relationship with organic matter (*t*_(10)_ = 2.07, *p* = .065) and a marginally negative relationship with minimum distance from the lake (*t*_(10)_ = −2.08, *p* = .064). Furthermore, the number of individuals in the traps increased slightly with distance from the lake (*t*_(10)_ = 2.36, *p* = .04).

**TABLE 3:** Average numbers of invertebrates in epibenthic traps and Shannon index with standard deviation and number of taxa in 18 Lava Caves near Lake Mývatn in Iceland.

Cave	Acarina	Clado	Cspha	Aharp	Mhirs	Alona	Colle	Cope
1	0.2 ± 0.41	0.0	0.3 ± 0.82	0.2 ± 0.41	0.0	0.0	0.0	20.3 ± 15.85
2	0.0	0.0	1.0 ± 1.67	0.3 ± 0.82	0.2 ± 0.41	0.0	0.0	14.7 ± 14.75
5	0.0	0.0	2.5 ± 3.21	0.0	0.2 ± 0.41	0.7 ± 1.21	0.0	26.2 ± 35.40
6	0.0	0.0	2.0 ± 0.00	0.0	0.5 ± 0.71	0.0	0.0	56.0 ± 45.25
7	0.3 ± 0.50	0.3 ± 0.50	2.8 ± 2.75	0.0	0.0	0.0	0.0	20.8 ± 17.73
10	0.3 ± 0.50	0.0	0.3 ± 0.50	0.0	0.0	0.0	0.0	35.0 ± 29.13
11	2.3 ± 4.50	0.0	1.0 ± 1.15	0.0	0.0	0.8 ± 1.50	0.0	4.0 ± 2.94
12	0.0	0.0	16.3 ± 11.90	0.0	0.3 ± 0.50	1.3 ± 2.50	0.0	93.0 ± 30.42
17	0.3 ± 0.58	0.0	3.0 ± 2.00	0.0	0.3 ± 0.58	0.0	0.3 ± 0.58	11.3 ± 11.37
18	0.3 ± 0.50	0.0	18.3 ± 26.41	0.0	0.0	0.0	0.0	29.8 ± 32.26
19	0.0	0.0	2.3 ± 1.50	0.0	0.0	0.0	0.3 ± 0.50	4.8 ± 2.87
20	0.0	0.0	14.8 ± 13.68	0.8 ± 1.30	0.0	0.2 ± 0.45	0.0	39.6 ± 24.04
21	0.0	3.0 ± 5.20	26.3 ± 2.31	0.0	0.0	0.0	0.0	28.0 ± 17.52
22	0.0	0.5 ± 31.00	7.3 ± 6.40	0.5 ± 0.58	0.0	0.0	0.0	11.5 ± 3.87
23	0.0	2.0 ± 3.37	6.0 ± 6.98	2.5 ± 3.79	0.3 ± 0.50	0.0	0.0	33.3 ± 12.45
25	0.0	0.0	4.0 ± 7.27	0.0	0.0	0.3 ± 0.82	0.0	45.7 ± 39.14
27	0.3 ± 0.50	0.0	20.8 ± 25.24	0.0	0.0	0.0	0.0	32.3 ± 18.84
17B	0.0	0.0	7.7 ± 10.02	0.0	0.0	0.0	0.0	33.3 ± 33.61

Cave	Nema	Ostra	Oligo	Roti	# individuals	Shannon	Taxa #
1	0.0	0.2 ± 0.41	0.0	81.5 ± 65.93	103.8 ± 72.87	0.54 ± 0.162	6
2	0.0	0.7 ± 0.82	0.0	7.3 ± 7.09	26.7 ± 23.25	0.72 ± 0.381	6
5	0.0	0.2 ± 0.41	0.0	32.7 ± 31.17	69.3 ± 69.92	0.73 ± 0.272	6
6	0.0	1.0 ± 1.41	0.0	149.5 ± 119.50	215.5 ± 167.58	0.68 ± 0.011	5
7	0.0	0.3 ± 0.50	0.0	15.3 ± 12.12	46.7 ± 32.26	0.76 ± 0.513	6
10	0.0	0.3 ± 0.50	0.0	9.3 ± 12.28	55.0 ± 24.44	0.50 ± 0.261	5
11	0.0	1.0 ± 1.41	0.0	9.3 ± 11.00	29.2 ± 11.29	0.90 ± 0.309	6
12	0.0	1.5 ± 1.73	0.0	21.3 ± 6.65	145.5 ± 25.85	0.86 ± 0.225	6
17	0.0	1.0 ± 1.00	0.0	67.7 ± 54.45	101.7 ± 53.15	0.65 ± 0.223	7
18	0.0	0.5 ± 1.00	0.0	204.0 ± 189.24	323.0 ± 153.48	0.79 ± 0.409	5
19	0.0	1.0 ± 1.15	0.0	16.8 ± 17.21	44.2 ± 18.62	0.80 ± 0.226	5
20	0.2 ± 1.63	0.4 ± 0.55	0.0	32.0 ± 42.51	108.4 ± 70.80	0.99 ± 0.055	7
21	0.0	0.7 ± 1.15	0.0	9.0 ± 7.21	89.7 ± 20.31	1.10 ± 0.310	5
22	0.0	1.3 ± 1.26	0.3 ± 0.50	37.0 ± 25.78	82.0 ± 24.91	1.08 ± 0.257	6
23	0.0	0.3 ± 0.50	0.0	55.0 ± 37.16	129.5 ± 57.10	1.03 ± 0.273	7
25	0.7 ± 1.63	0.5 ± 0.55	0.0	17.8 ± 10.57	163.3 ± 123.05	1.00 ± 0.202	6
27	0.0	0.0	0.0	57.0 ± 54.39	142.0 ± 74.97	0.98 ± 0.223	4
17B	0.0	0.3 ± 0.58	0.0	80.3 ± 127.12	122.3 ± 134.74	0.57 ± 0.414	4

*Note*: Some taxa names have been shortened: Clado (Cladocera sp), Cspha (*Chyodorus sphaericus*), Aharp (*Acroperus harpae*), Mhirs (*Macrothrix hirsuticornis*), Alona (Alona sp), Colle (Collembola), Cope (Copepoda), Nema (Nematoda), Ostra (Ostracoda), Oligo (Oligochaeta), Roti (Rotifera).

**FIGURE 6:**
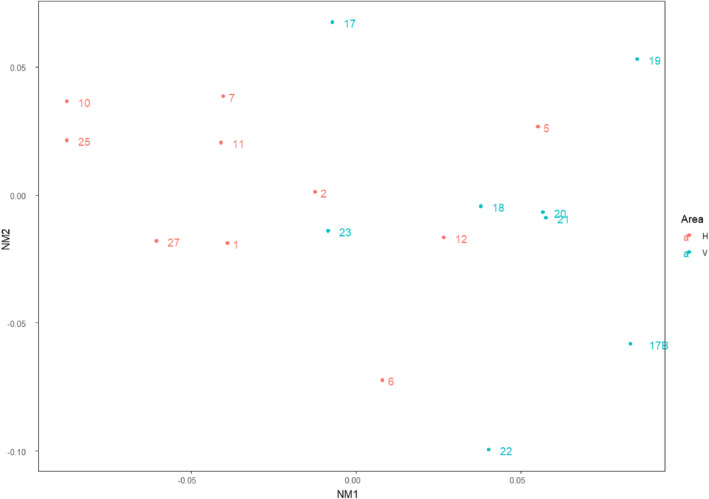
NMDS ordination showing the relationship among epibenthic invertebrate communities in lava cave ponds. Caves from the two areas are shown, Haganes (H) in red and Vindbelgur (V) in blue. No environmental variables were found to significantly relate to the invertebrate communities and are thus not shown.

**FIGURE 7:**
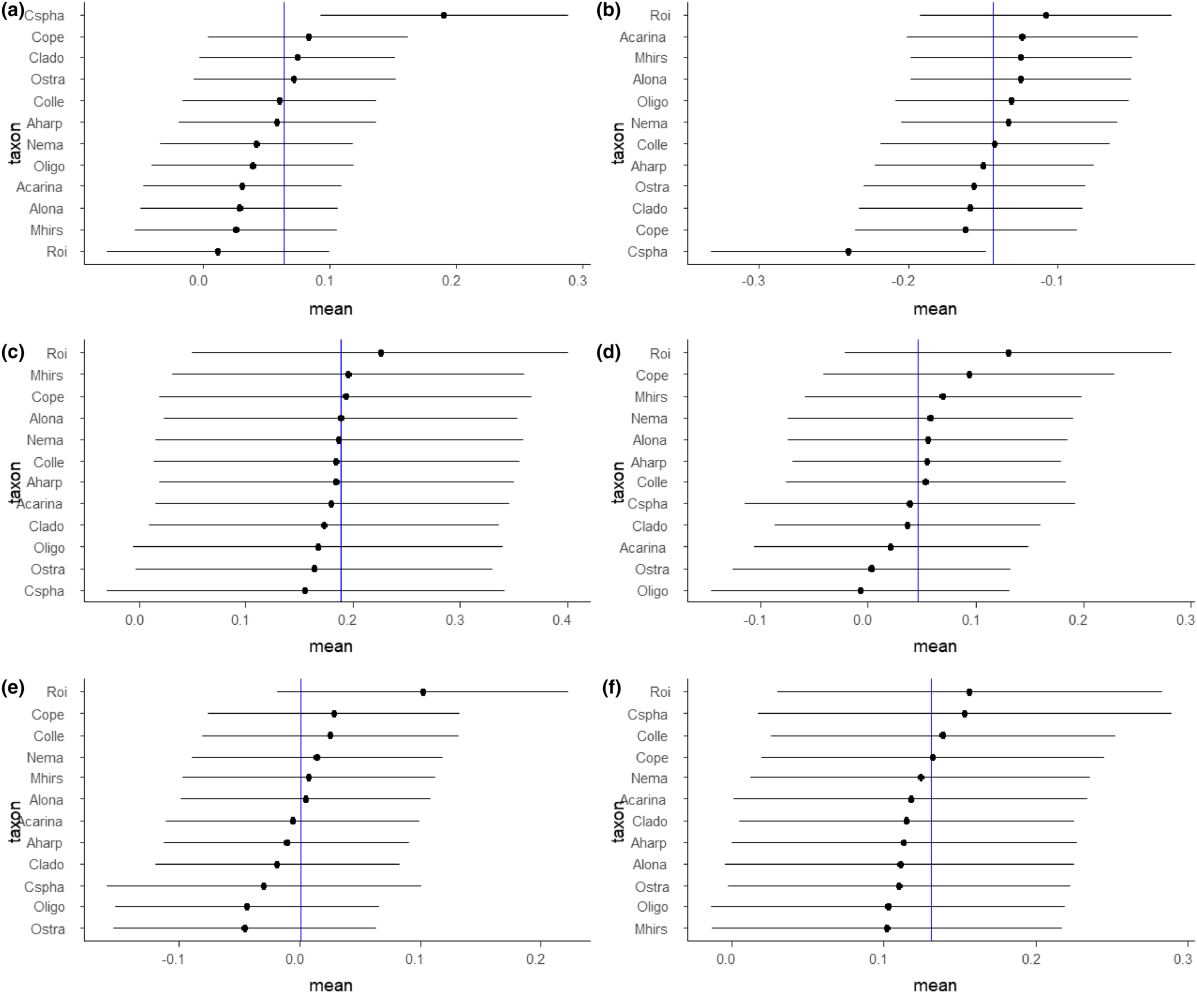
Mean responses, with standard error, of different taxa from epibenthic invertebrate communities in lava cave ponds in relation to the average fixed effect estimate from a linear mixed model studying the effect of environmental variables on invertebrate communities with stones nested within caves as a random factor. The responses were obtained by parametric bootstrapping. The environmental variables are as follows: Organic matter (a), pH (b), conductivity (c), distance to Lake Mývatn (d), temperature (e), and oxygen concentration (f). Abbreviations for species are explained in Table 3.

The simplest solution for NMDS analysis was five dimensional, which gave a stress of 0.080 (Figure 6). None of the environmental variables were clearly influencing the invertebrate communities. There was some separation between the two areas along NMDS axis 1. According to the PERMANOVA, community composition varied the most strongly with the amount of organic matter (*F*_(1.68)_ = 2.7, *p* = .006, *R* ^2^ = .03), followed by pH (*F*_(1.68)_ = 2.3, *p* = .009, *R* ^2^ = .03), and conductivity (*F*_(1.68)_ = 2.2, *p* = .007, *R* ^2^ = .03). However, our LMMs indicated that ecological variables had little effect across taxa. The closest to being significant was pH (*F*_(1,9.4)_ = 4.6, *p* = .06). We then examined the responses across the different taxa, where we did not get any statistically clear results, as reflected in plots of responses across the different taxa in relation to environmental factors (Figure 7a–f). However, *C. sphaericus* (Cladocera) the most common and abundant taxon, had a higher than average response to organic matter and lower than average to pH.

## 4 DISCUSSION

Both the benthic and epibenthic invertebrate communities in these caves are species poor with only 26 taxa found in the stone samples and 12 in the epibenthic samples. It is likely that those two communities are shaped by different factors. Indeed, we found that the availability of organic matter was a major driver of overall abundance and composition of the benthic communities, while pH and conductivity had more modest effects. This suggests that niche‐based processes were important for structuring the benthic communities. In contrast, none of the environmental variables we examined appeared to structure the epibenthic communities. While we cannot rule out the importance of niche‐based processes for the epibenthic communities altogether, our results are consistent with the possibility that the epibenthic communities are more strongly influenced by neutral processes such as colonization dynamics. Furthermore, we cannot rule out that the taxonomic resolution we chose influenced our results and in higher‐order groups, we may have had taxa with different ecological requirements, resulting in increased noise in the results.

In addition to being species poor, these communities have low density of invertebrates, compared to other Icelandic spring‐fed freshwater systems (Govoni et al., 2018; Kreiling et al., 2021, 2022). A further striking observation, in comparison to other Icelandic freshwater systems, was the low densities of Chironomidae. Chironomidae are usually the most dominant invertebrate group in all Icelandic freshwater systems (Hrafnsdóttir, 2005), rivers, lakes, and springs (Govoni et al., 2018; Kreiling et al., 2018). The cave ponds studied here seem to be extremely oligotrophic, where the size, and perhaps the orientation of the cave openings determine area available for primary production and the amount of organic material falling into the caves. These two interrelated factors had a clear relationship with the density and communities of benthic invertebrates in the caves. It was clear that some taxa benefitted from high nutrient input, for example, some Cladocera taxa, Ostracoda, and Tanytarsini. Where nutrient input was low, taxa such as Tardigrada, *Macrothrix hirsuticornis*, and Orthocladiinae occurred in higher densities. The polarization between these two Chironomidae subfamilies was interesting and was seen in their response to all the environmental variables, which may be an indicator of different evolutionary histories, reflected in different life history strategies.

It is likely that an important factor in shaping the invertebrate communities in these cave ponds is the presence of a top predator, the Arctic charr, which is found in all the caves studied. The charr in these caves are opportunistic feeders who take both food falling on the surface of the caves as well as actively hunting benthic and epibenthic prey (Kristjansson et al., 2012, Árnason et al., in prep). As selective visual predators, charr may thus keep down various populations of invertebrates, which they prefer to eat, and prevent the colonization of new taxa into the cave systems (Fukami, 2015). Such top‐down influences have been seen before, for example, in how perch (*Perca fluviatilis*) influenced invertebrate communities in a small pond experiment (Diehl, 1992). This, however, needs further studying. Another interesting finding from the experiment of Diehl (1992) was the influence of habitat structure on the invertebrate communities, where vegetation, and thus increased habitat structural complexity, increased the biomass of invertebrates. The cave ponds have a complex lava structure near the cave openings and then a flat soft mud bottom, which in some cases is covered by an algal mat. These muddy areas of the caves have extremely low diversity and density of invertebrates in both the muddy bottom (Kristjánsson et al. unpublished results) and in epibenthic communities (Combot, 2018). The more complex lava structure by the opening may thus create a refugium for the invertebrates where they are safe from predators, but at the same time limit the available space for the communities.

The size of the ponds, especially the available habitat at the shoreline, may be a key for the low taxa richness of the invertebrate communities, especially when it comes to the epibenthic habitat. It is thus perhaps not a surprise that we do not find support for niche‐based processes being important drivers of the epibenthic community structure. Small populations are more likely to be strongly influenced by various factors and go extinct, because of bad luck or competitive exclusion by another species within the community (Orrock & Fletcher, 2005). It may also be that some species are well established in the caves, for example, *Chydorus sphaericus* and Orthocladiinae, which were found in all the caves, while other taxa are more temporarily unstable, for example, Ostracoda, where there was a great diversity among caves, and many caves housed unique species, when compared to other caves (Alalaj et al., 2019). These taxa then regularly re‐colonize the caves, where the order of the colonizers may be important (Fukami, 2015). Here, it is important to consider the ease different species have for colonization, with flying insects (e.g., Chironomidae) being generally better colonizers than crustaceans, who rely on resting eggs or direct water connections for dispersal. In this respect, it would be interesting to see how temporally stable the cave pond communities are, both within and among years. In another spring‐fed system in Iceland where Arctic charr is present as a top predator, there are considerable seasonal changes in the invertebrate community (Kreiling et al., 2021).

The order and frequency of species colonization in the ponds may be important (Fukami, 2015). We had predicted that we could see relationships between the invertebrate communities and the distance from Lake Mývatn, which is likely the main source of origin for invertebrates found in the caves. We did not find such a relationship. This may be because the source for the invertebrate communities in the ponds may be manifold, as many ponds and small lakes can be found in the lava systems around Lake Mývatn, especially close to the Vindbelgur area. Furthermore, the invertebrates may use groundwater channels to enter the caves, where groundwater flow does not necessarily relate to distances to Lake Mývatn or nearby waterbodies.

On a large scale, the two areas differ in the main direction of groundwater flow. In the Haganes area, the main groundwater flow is from east to west, from the lake toward river Laxá, while in the Vindbelgur area groundwater is flowing from north‐west to south‐east, where there are numerous ponds and lakes, and toward Lake Mývatn (Ólafsson, 1979). However, at a finer scale, the exact groundwater flow is not well known. This has resulted in these two areas differing in important ecological factors like conductivity and oxygen saturation. We would have predicted that the invertebrate communities would differ between these two areas, as we have observed differences in important life history characters of Arctic charr populations from these different areas (Judson et al., 2024; Leblanc et al., 2024), but did not. All this taken together may call for a more detailed study on the origin of the invertebrates found in these caves.

In the benthic communities, we did find that niche‐based processes may be important for shaping the invertebrate communities. The most important of those being availability of energy, as discussed above. Another important factor was pH, where we saw fewer taxa in communities with higher pH, which was further reflected in different preferences of taxa toward differences in pH. Previous studies have shown pH to be important for community structure of Copepoda (Pipan et al., 2006), and we saw some indications that there were higher number of Copepoda at higher pH. We had predicted that temperature would be an important driver of the community structure, as has been seen in other spring‐fed systems in Iceland (Govoni et al., 2018; Kreiling et al., 2018, 2021, 2022), but did not find clear support for this beside seeing an increased number of taxa at higher temperature. These results may reflect the small variation in temperature we observed, with only 1.9°C difference between the highest and lowest.

Ponds are good model systems for research in ecology and evolutionary biology (De Meester et al., 2005). The ponds studied here are numerous and spread over a few km^2^—we only studied a small proportion of available ponds. Here it is clear that the invertebrate communities of these lava cave ponds are shaped by an interaction of neutral‐ and niche‐based processes, where the availability of energy is likely to be a key factor. This could be further studied by developing experimental manipulations of these systems. Furthermore, we have been monitoring the ponds described here since 2013, where we twice annually collect samples of invertebrate communities on hard bottom along with our study of the Arctic charr in the ponds. This gives us opportunities to look at temporal stability in the invertebrate communities providing further understanding of how neutral‐ and niche‐based processes work together in shaping invertebrate communities. Furthermore, these ponds represent unique ecosystems that are extremely vulnerable to human disturbance, making it even more important to understand how their biodiversity is shaped and maintained. Which is especially timely, as such ecosystems currently have no special conservation status in Iceland.

## AUTHOR CONTRIBUTIONS

**Bjarni K. Kristjánsson:** Conceptualization (equal); formal analysis (equal); funding acquisition (equal); investigation (equal); methodology (equal); project administration (lead); writing – original draft (lead); writing – review and editing (lead). **Doriane Combot:** Conceptualization (equal); investigation (equal); methodology (equal); writing – original draft (equal). **Anett Reilent:** Investigation (equal); methodology (equal); writing – original draft (equal). **Joseph S. Phillips:** Formal analysis (equal); writing – original draft (equal); writing – review and editing (equal). **Camille A.‐L. Leblanc:** Conceptualization (equal); formal analysis (equal); investigation (equal); methodology (equal); project administration (equal); writing – original draft (equal); writing – review and editing (equal).

## CONFLICT OF INTEREST STATEMENT

The authors declare no conflict of interest in relation to this manuscript.

## Data Availability

Data are available in Dryad—https://doi.org/10.5061/dryad.jwstqjqhw.
